# The impacts of patient mistreatment on healthcare workers’ role behaviors: a study in Chinese Fangcang shelter hospitals

**DOI:** 10.1186/s12912-023-01574-5

**Published:** 2023-11-24

**Authors:** Wei Yan, Na Bao, Shuangjiang Zheng, Huan Wang, Dongmei Yue, Li Chen

**Affiliations:** 1 School of Economics and Business Administration, Chongqing University, Chongqing, China; 2 Department of Medical Affairs, The First Affiliated Hospital of Chongqing Medical University, Chongqing, China; 3 Development and Planning Department, Chongqing Medical University, Chongqing, China; 4 School of Medicine and Health Management, Tongji Medical College, Huazhong University of Science & Technology, Wuhan, China; 5 Department of Prevention and Health Care, SouthWest Hospital, Chongqing, China; 6 Hospital Administration Office, Chongqing University Central Hospital, Chongqing Emergency Medical Center, Chongqing, China

**Keywords:** Patient mistreatment, Emotional exhaustion, Service performance, Patient-oriented organizational citizenship behavior, Displaced aggression by patients

## Abstract

**Background:**

Fangcang shelter hospitals have played an important role in the battle against the COVID-19 epidemic in China. Patients’ verbal and physical attacks on medical workforce are prone to occur in such hospitals. This study explored the impacts of patient mistreatment on healthcare workers’ role behaviors (service performance and patient-oriented organizational citizenship behavior).

**Methods:**

We examined the influence of patient mistreatment on service performance and patient-oriented organizational citizenship behavior, as well as the mediating effect of emotional exhaustion and the moderating effect of displaced aggression by patients, using hierarchical linear regression and conditional process analysis.

**Results:**

Patient mistreatment was positively associated with emotional exhaustion among healthcare workers, while emotional exhaustion was negatively associated with service performance and patient-oriented organizational citizenship behavior. Mediation analysis revealed that emotional exhaustion mediated the association between patient mistreatment and both types of role behaviors. Moderated mediation analysis found that the mediation effect was weaker when the displaced aggression by patients was high.

**Conclusions:**

The findings clarified the relationship among patient mistreatment, emotional exhaustion, service performance, and patient-oriented organizational citizenship behavior. Additional assistance should be provided to healthcare workers dealing with patient mistreatment. Displaced aggression by patients attenuates the positive effects of patient mistreatment on the emotional exhaustion of healthcare workers. Our findings reveal the mechanism and boundary conditions of patient mistreatment affecting healthcare workers' service performance and patient-oriented organizational citizenship behavior.

## Background

Workplace violence against healthcare workers has been on the rise since the COVID-19 pandemic [1]. Assaults against healthcare workers are increasing at an alarming rate globally, especially verbal attacks by patients [2, 3]. In 2022, omicron virus led to the pandemic outbreak of neo-coronavirus pneumonia in Shanghai, with mass infected patients arising nationwide in a short period of time. Fangcang shelter hospitals were quickly established and used to isolate and treat patients with mild to moderate COVID-19. Such hospitals were large-scale, temporary hospitals built by converting public venues such as stadiums and exhibition centers into healthcare facilities. Healthcare professionals in Fangcang shelter hospitals also suffer from patient mistreatment. Patients who enter the concentration isolation sites of these hospitals are more likely to suffer from mental health problems, such as anxiety, depression, anger, etc., because they are in a relatively narrow space [4]. This may cause patients to act aggressively toward the healthcare staff. Numerous studies have shown that workplace violence adversely affects the quality of patient care and reduces the organizational commitment, job performance, and job satisfaction of healthcare workers [5–8]. Patient mistreatment negatively affects the role behaviors of healthcare workers [9]. However, there are few studies on how and when this occurs. Therefore, the current research aims to clarify the relationship between patient mistreatment and healthcare workers' role behaviors. Specifically, we focus on two types of role behaviors: service performance and patient-oriented organizational citizenship behavior (OCB hereafter). Effective interventions are necessary to support healthcare workers during health emergencies and possible future outbreaks.

Previous studies have elaborated on the negative impact of customer mistreatment on employees, and it is important to explore the boundary conditions behind it. For example, accountability and supervisor support can weaken the impact of customer mistreatment on employees' negative emotions [10, 11]. At present, there is no perspective from the self-verification of healthcare workers to explore the boundary conditions that alleviate the negative effects of patient mistreatment. Based on the self-verification theory, healthcare professionals constantly accept and process external information to enhance their self-concept. This study proposes that displaced aggression by patients (DAP hereafter) may have a moderating effect between patient mistreatment and emotional exhaustion. Healthcare professionals with extensive work experience are able to detect changes in patient behaviors and emotions during this emergency epidemic, while standing in the patient's perspective to think about problems and empathize with the patient. The patients "indiscriminate" aggression may stimulate empathy among healthcare workers, reducing the depletion of their own self-efficacy and emotional resources, thereby influencing service performance and patient-oriented OCB.

Therefore, we aimed to study the impacts of patient mistreatment on the service performance and patient-oriented OCB of healthcare workers and explore the mechanisms and boundary conditions behind this relationship. This study attempted to answer the following questions: Is patient mistreatment related to service performance and patient-oriented OCB through emotional exhaustion? Can healthcare professionals perceive DAP as a protective resource to reduce emotional exhaustion?

### Patient mistreatment and emotional exhaustion

Customer mistreatment refers to unfair and low-quality interpersonal treatment of service personnel by customers during the service process, including cruel hostility, insult, and other acts of intensity less than physical violence [12]. Walker et al. (2014) found that customer mistreatment as a source of stress can induce negative emotions in employees [13]. Customer mistreatment indirectly affects the organization’s negative reputation through employees’ OCB [14]. Specifically, in the healthcare service industry, customer mistreatment is referred as patient mistreatment. However, there are limited academic studies on the impact of patient mistreatment on healthcare workers, particularly in Fangcang shelter hospitals.

According to conservation of resource theory, stress results from circumstances involving threatened or actual loss of valued resources, and individuals are motivated to defend, conserve, and acquire the resources they value [15]. Being judged or criticized by others (such as leaders, customers or patients) would lead to a loss of resources that are valuable in meeting job demands. Especially for healthcare workers, serving patients is itself a process of consuming physical and psychological resources [16–18]. Healthcare workers are suffering anxiety due to the heavy workloads in Chinese Fangcang shelter hospitals, which has depleted a great deal of the healthcare professionals' resources to perform their duties [19]. The occurrence of patient mistreatment is not only unable to replenish the original emotional resources of healthcare providers but also requires them to consume additional emotional resources to cope with it [20, 21]. If healthcare professionals’ existing resource reserves are repeatedly depleted without being replenished, it may provoke harmful emotional and psychological reactions, such as emotional exhaustion. In addition, patients' rude and aggressive behavior would impose a greater demand on healthcare professionals who need to spend more resources to regulate their emotions just in order to sustain a normal doctor-patient relationship [22–24]. For example, the established organizational rules or regulations require healthcare workers to suppress their overt expressions of negative emotions. As a result, healthcare professionals would have to exercise more energy to regulate their emotions, which would lead to an overuse of valuable resources, resulting in emotional exhaustion [25]. Based on the above analyses, the following hypothesis is proposed:

- H1: Patient mistreatment is positively associated with emotional exhaustion of healthcare workers.

### The mediating role of emotional exhaustion

In a service environment characterized by uncertainty and interdependence, employees need to go beyond the narrow scope of work to serve customers [26]. The distinction between in-role and extra-role behavior is reflected in the discretionary and formalization of employee service behaviors [27]. In-role behavior refers to the organization’s members’ activities within the provisions that must be performed to receive the corresponding remuneration [28]. Essentially, it belongs to the employees’ daily work tasks, usually for service performance [29]. The extra-role behavior is the spontaneous initiative of employees, which transcends the role and organizational remuneration system [30], such as autonomous work effort and OCB.

Resources are a set of important factors that can help individuals achieve their goals or meet their psychological needs [31]. Faced with customer mistreatment, employees must consume additional resources to engage in effective self-motivation to maintain good performance [32]. Studies have shown that emotional exhaustion during COVID-19 has a significant negative effect on unhealthy diets of healthcare workers [33]. In the case of emotional exhaustion, healthcare professionals will be more inclined to reduce the input of other resources, such as reducing work input or engagement, to compensate for the resource loss [34]. Due to the loss of resources, workers may spend time and attention calming themselves down, which reduces their involvement and attention at work and leads to a decline in service performance [35]. Healthcare professionals who have lost resources due to the increasing job stress from patient mistreatment, will be more protective of existing resources to prevent further loss. Thus, they may reduce their commitment and responsibility to patients by reducing patient-oriented OCB [36]. In general, patient mistreatment leads to a loss of emotional resources, which may further lower the initiative and motivation of healthcare professionals to provide services, thereby reducing their interests and willingness to serve patients [37]. Accordingly, we propose the following hypotheses:

- H2a: Emotional exhaustion is negatively related to service performance.
- H2b: Emotional exhaustion is negatively related to patient-oriented OCB.
- Combining H1 and H2, this study proposes the following hypotheses regarding the mediating effects of emotional exhaustion:
- H3a: Emotional exhaustion mediates the association between patient mistreatment and service performance.
- H3b: Emotional exhaustion mediates the association between patient mistreatment and patient-oriented OCB.

### The moderating role of displaced aggression by patients

Dodge et al. (1987) considered reactive aggression as an individual's defensive response to anger when confronted with frustration [38]. DAP is a particular type of reactive aggression. To eliminate anger, the attacked person will try to retaliate against the attacker [39]. However, they are often unable or unwilling to attack the attacker (e.g., their supervisor), then they often turn to attacking other innocent parties [40]. For example, workers show negative emotional and behavioral reactions toward their families after feeling dissatisfied with their leader [41], and studies have also shown that work-family conflicts can lower employees’ emotions and even engage in aggressive behavior toward those around them [42].

Based on the analysis above, we attempted to incorporate displaced aggression as a boundary condition to explore ways to mitigate the negative effects of patient mistreatment. According to self-verification theory, individuals in the process of self-concept formation will continuously receive, integrate, interpret, and modify external information, thereby affecting their self-concept [43, 44]. Self-verification theory emphasizes how healthcare professionals see themselves and what they see from their patients [45]. In order to maintain self consistency, they exhibit behaviors that are consistent with their self-concept [46]. On the one hand, it is the responsibility of healthcare workers to provide high-quality medical services to patients and care for them [47]. Their professional identity is also characterized by kindness and care [48]. Healthcare workers tend to view being mistreated as a failure of service, thereby reducing their self-esteem and self-concept [49]. However, when workers witness patients frequently expressing anger toward themselves, other healthcare providers, and/or shelter hospital managers, they may catch those signals through their empathic abilities. Previous studies have found that healthcare professionals with high empathy skills are more likely to interpret patients' "indiscriminately" attacks as a result of their dilemma, rather than intentionally mistreating others [10]. We predict that when healthcare workers encounter DAP, they tend to regulate their emotions with a high level of care due to seeking consistence between environment and their self-concept. On the other hand, self verification assessments can also reduce emotional exhaustion by confirming a person's self-concept [50]. People hope to reduce uncertainty about their self-concept and ensure that they maximize their enthusiasm for self-awareness [51]. Subsequently, they may construct a personal understanding of the mistreatment and build an interpretation that patients "indiscriminately" attack people around because of anxiety, nervousness, or depression rooted in the treatment of coronavirus disease in Fangcang shelter hospitals. Being mistreated by patients should not be attributed to the workers’ own professional and technical competence, thus reducing the risk of depletion of their self-concept and the consumption of their own resources. Accordingly, we propose the following hypothesis:

- H4: DAP moderates the relationship between patient mistreatment and emotional exhaustion such that the relationship is weaker when DAP is high versus low.

Combining H3a, H3b and H4, we propose a moderated mediation model of DAP. If the mediation hypotheses H3a, H3b and the first-stage moderation hypothesis H4 hold, it is reasonable to expect indirect effects of patient mistreatment on role behaviors through emotional exhaustion would also vary according to the different levels of DAP. Therefore, we put forward the following hypotheses:

- H5a: DAP moderates the indirect effect of patient mistreatment on service performance through emotional exhaustion such that the indirect effect is weaker when DAP is high versus low.
- H5b: DAP moderates the indirect effect of patient mistreatment on patient-oriented OCB through emotional exhaustion such that the indirect effect is weaker when DAP is high versus low.

**Fig. 1:**
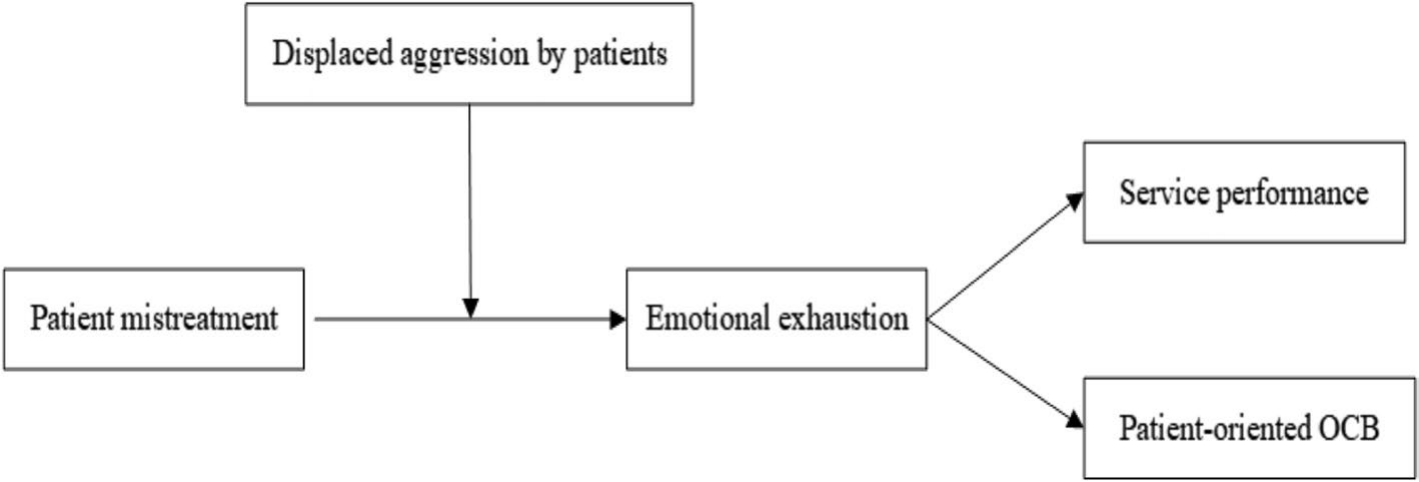
The proposed moderated mediation model

We summarize our theoretical model in Fig. 1.

## Methods

### Participants and procedure

The online survey was administered through a questionnaire website (https://www.wjx.cn/), with the link delivered to qualified participants with the help of medical team leaders and nursing team leaders in Fangcang Shelter Hospital in December 2022. To prevent unnecessary administrative coercion, each questionnaire was anonymized, and each IP address could only be submitted once. The sample consisted of medical and nursing staff from the Yinglong, Yutong, Yuelai, and Nanping shelter hospitals. In total, 639 questionnaires were distributed. After excluding invalid questionnaires that failed to pass the attention check, 493 valid responses were obtained. The demographic characteristics of the participants are presented in Table 1.

**Table 1:** Sociodemographic characteristics (*N* = 493)

Variables	N	%
**Gender**		
Male	84	17.0
Female	409	83.0
**Age**		
20–29	136	27.6
30–39	289	58.6
40–49	64	13.0
≥ 50	4	0.8
**Working years**		
< 5	108	21.9
6–15	293	59.4
16–25	76	15.4
≥ 26	16	3.2
**Marital Status**		
Unmarried	139	28.2
Married	335	68.0
Divorce	19	3.9
**Education**		
Specialized and below	60	12.2
Undergraduate	358	72.6
Master	53	10.8
PhD	22	4.5
**Technical title**		
No title	25	5.1
Junior	218	44.2
Intermediate	219	44.4
Deputy Senior	28	5.7
Senior	3	0.6
**Professional category**		
Medical	93	18.9
Nursing	381	77.3
Medical Technology	5	1.0
Administration and Others	14	2.8

### Measures

A back-translation procedure was conducted to ensure the accuracy and consistency of the translated scales [52]. A bilingual management professor translated items into Chinese from the original English, and then another management professor translated items from Chinese back into English. Discrepancies were checked, discussed, and finalized by both professors. The scales used in this study have been widely applied in academic research. The original scales were modified appropriately to fit the research situation. The specific scales were as follows:

- Patient mistreatment was measured by a 5-item scale (1 = never to 5 = always) developed by Shao et al. (2014) [53]. Participants were asked, “How often over the past month have you had the following occur from a patient?” Sample items included “said inappropriate things.” Cronbach's α for the scale was 0.929.
- Emotional exhaustion was measured by a 6-item scale (1 = strongly disagree; 5 = strongly agree) developed by Aryee et al. (2008) [54]. Sample items included “I feel emotionally drained from my work”. Cronbach's α for the scale was 0.931.
- Service performance was measured by a 5-item scale (1 = strongly disagree; 5 = strongly agree) developed by Bettencourt et al. (1997) [55]. Sample items included “I can perform all those tasks for patients who are required of me”. Cronbach's α for the scale was 0.936.
- Patient-oriented organizational citizenship behavior was also measured by a 5-item scale (1 = strongly disagree; 5 = strongly agree) developed by Bettencourt et al. (1997) [55]. Sample items included “I can voluntarily assist patients even if it means going beyond job requirements.” Cronbach's α for the scale was 0.891.
- Displaced aggression by patients was measured by a 5-item scale (1 = strongly disagree; 5 = strongly agree) developed by Denson et al. (2006) [56]. and we selected the top three items with the highest factor loadings. Considering that the questionnaire was answered by healthcare workers, a reference-shift method was adopted to modify the subject in each item following Schuster et al. (2022) [57]. Sample items included “When someone or something makes the patient angry, he or she is likely to take it out on another person”. Cronbach's α for the scale was 0.917.

Gender, age, working years, education, technical title, working weeks in shelter hospitals and daily working hours were used as covariates.

### Statistical analyses

Statistical analyses were conducted using SPSS 26.0 and Mplus 8.3, and descriptive statistics were computed for all variables. Considering common method variance (CMV hereafter) that might bias our findings, we introduced a variable that is completely unrelated to this study in theory as a marker variable [58], namely, software self-efficacy [59, 60]. This variable was measured on a 3-item scale. Sample items included “I could complete the job using the software package if there was no one around to tell me what to do as I go.” Cronbach's α for the scale was 0.834. In addition, we tested the hypotheses using hierarchical regression analysis, bootstrap test and conditional process analysis (specifically, moderated mediation in this study).

## Results

As shown in Table 2, the five-factor model was better than the other models, which indicates that the model fits best (χ2/df = 2.961, TLI = 0.947, CFI = 0.954, RMSEA = 0.063, SRMR = 0.04) and that the variables have reasonable discriminant validity.

**Table 2:** Confirmatory factor analyses

Model	χ2	df	χ2/df	TLI	CFI	RMSEA	SRMR
Five-factor (PM, DAP, EE, SP, OCB)	716.623	242	2.961	0.947	0.954	0.063	0.040
Four-factor (PM, DAP, EE, SP + OCB)	1676.381	246	6.815	0.843	0.860	0.109	0.086
Four-factor (PM + DAP, EE, SP, OCB)	1715.306	246	6.973	0.839	0.856	0.110	0.079
Three-factor (PM + DAP, EE, SP + OCB)	2673.217	249	10.736	0.737	0.763	0.141	0.109
Three-factor (PM + EE, DAP, SP + OCB)	3445.468	249	13.837	0.654	0.687	0.161	0.133
Two-factor (PM + DAP + EE, SP + OCB)	4406.433	251	17.556	0.553	0.594	0.183	0.146
One-factor (PM + DAP + EE + SP + OCB)	7501.592	252	29.768	0.224	0.291	0.242	0.232

*N* = 493, *PM* patient mistreatment, *DAP* displaced aggression by patients, *EE* emotional exhaustion, *SP* service performance, *OCB* patient-oriented organizational citizenship behavior

We used the Harman single-factor test to assess CMV. Specifically, the results of exploratory factor analyses showed that the first principal component explained 29.47% of the variance. This value was significantly less than the 50% benchmark, suggesting no serious problems with the CMV. This study tests CMV by controlling for software self-efficacy to compare the difference between the bias correlation coefficient and its zero-order correlation coefficient [61]. As is shown in Table 3, the correlation coefficients between the variables remained significant after controlling for the marker variable, indicating that the CMV problem was not severe in the current study.

**Table 3:** Descriptive statistics and correlations

Variables	Mean	SD	1	2	3	4	5
PM	1.609	0.749	(0.929)	0.379***	0.409***	-0.038	-0.015
DAP	3.291	1.107	0.381***	(0.917)	0.304***	0.000	0.147**
EE	2.481	0.965	0.416***	0.305***	(0.931)	-0.107*	-0.121**
SP	4.346	0.633	-0.037	0.132**	-0.173***	(0.936)	0.489***
OCB	3.773	0.733	-0.061	-0.012	-0.166***	0.527***	(0.891)
SSE	3.849	0.708	-0.084	0.041	-0.227***	0.257***	0.292***

The coefficients below the diagonal line indicate the correlation coefficients. The numbers on the diagonal line represent Cronbach's α coefficients. The coefficients above the diagonal line indicate a partial correlation coefficient after controlling for the marker variable

*PM* patient mistreatment, *DAP* displaced aggression by patients, *EE* emotional exhaustion, *SP* service performance, *OCB* patient-oriented organizational citizenship behaviour, *SSE* software self-efficacy

^*^p < 0.05

^**^p < 0.01

^***^p < 0.001 (two-tailed)

Table 3 presents the descriptive statistics and correlations among the variables. Patient mistreatment and emotional exhaustion were positively correlated (*r* = 0.416, *p* < 0.001). Emotional exhaustion was negatively correlated with service performance (*r* = -0.173, *p* < 0.001) and patient-oriented OCB (*r* = -0.166, *p* < 0.001).

### Testing for the mediating effect

We used hierarchical regression method to test the main effects [62]. As shown in Table 4, patient mistreatment was positively associated with emotional exhaustion (β = 0.526, *p* < 0.001), and emotional exhaustion was negatively associated with both service performance (β = -0.105, *p* < 0.01) and patient-oriented OCB (β = -0.095, *p* < 0.05). Thus, H1 and H2 are supported.

**Table 4:** Mediating effect of emotional exhaustion

Variables	EE		SP			OCB		
	M1	M2	M3	M4	M5	M6	M7	M8
Gender	0.100	0.186	0.146	0.148	0.167*	0.095	0.093	0.110
Age	-0.013	-0.015	-0.003	-0.003	-0.004	-0.005	-0.005	-0.007
Working years	-0.009	0.004	0.012	0.012	0.012	0.018	0.018	0.018
Education	0.020	0.008	0.029	0.028	0.029	0.069	0.069	0.070
Technical title	0.164	0.144	0.062	0.061	0.076	0.003	0.003	0.017
Working weeks in hospitals	-0.006	-0.008	-0.006	-0.006	-0.007	-0.012	-0.012	-0.013
Daily working hours	0.051	0.057*	0.012	0.012	0.018	0.016	0.016	0.021
SSE	-0.298***	-0.249***	0.239***	0.240***	0.214***	0.307***	0.306***	0.283***
PM		0.526***		0.011	0.066		-0.015	0.035
EE					-0.105**			-0.095*
R^2^	0.069	0.229	0.100	0.100	0.120	0.109	0.109	0.121
** F**	4.475**	15.926***	6.714***	5.967***	6.548***	7.402**	6.582*	6.647***

M1 to M2 indicates the application of the hierarchical regression to investigate the impact of independent variable PM on the mediating variable EE. M3 to M5 and M6 to M8 aim to investigate the impact of the mediator EE on the outcome variables SP and OCB, respectively

*PM* patient mistreatment, *EE* emotional exhaustion, *SP* service performance, *OCB* patient-oriented organizational citizenship behaviour, *SSE* software self-efficacy

^*^p < 0.05

^**^p < 0.01

^***^p < 0.001 (two-tailed)

Referring to the mediation analysis steps proposed by Zhao et al. (2010) [63], Table 5 shows the results of the mediating analysis using 5,000 bootstraps. The indirect effect of patient mistreatment on service performance was -0.055, and the confidence interval was (-0.093, -0.019), which did not include the value of zero, indicating that emotional exhaustion had a mediating effect. Similarly, emotional exhaustion mediated the impact of patient mistreatment on patient-oriented OCB. Thus, both H3a and H3b are supported.

**Table 5:** Bootstrap test for mediating effect

		**Effect**	**BootSE**	**BootLLCI**	**BootULCI**
PM-EE-SP	Indirect effect	-0.055	0.019	-0.093	-0.019
	Direct effect	0.066	0.041	-0.014	0.146
	Total effect	0.011	0.037	-0.062	0.084
PM-EE-OCB	Indirect effect	-0.050	0.022	-0.094	-0.007
	Direct effect	0.035	0.047	-0.057	0.127
	Total effect	-0.015	0.043	-0.099	0.069

*PM* patient mistreatment, *EE* emotional exhaustion, *SP* service performance, *OCB* patient-oriented organizational citizenship behavior

### Testing for the moderating effect

Hierarchical linear regression analysis was performed to test the moderating effect of DAP. Table 6 illustrates the moderating effects of DAP. DAP weakened the impact of patient mistreatment on emotional exhaustion (β = -0.107, *p* < 0.05).

**Table 6:** Moderating effect of DAP

Variables	EE		
	**M9**	**M10**	**M11**
Gender	0.100	0.155	0.172
Age	-0.013	-0.016	-0.016
Work years	-0.009	0.006	0.007
Education	0.020	-0.011	0.007
Technical title	0.164	0.130	0.119
working weeks in hospitals	-0.006	-0.011	-0.01
Daily working hours	0.051*	0.060*	0.056*
SSE	-0.298***	-0.249***	-0.246***
PM		0.445***	0.530***
DAP		0.146***	0.123**
PM*DAP			-0.107*
**R**^**2**^	0.069***	0.253***	0.260*
**F**	4.475***	16.284***	15.336***

*PM* patient mistreatment, *DAP* displaced aggression by patients, *EE* emotional exhaustion, *SSE* software self-efficacy

^*^p < 0.05

^**^ *p* < 0.01

^***^ *p* < 0.001 (two-tailed)

**Fig. 2:**
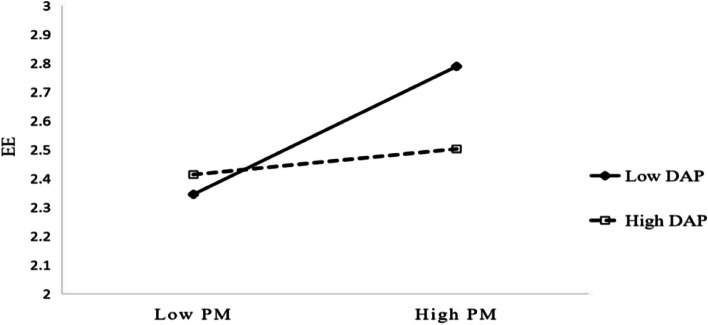
Moderating effect of DAP on the PM–EE relationship. Note: PM, patient mistreatment; DAP, displaced aggression by patients; EE, emotional exhaustion

Figure 2 shows that for high DAP, patient mistreatment has a weaker positive impact on emotional exhaustion. Thus, H4 is supported.

### Testing for the moderated mediation model

We further tested the moderating mediation effect on the indirect path using conditional process analysis. As shown in Table 7, when service performance was the dependent variable, the bootstrap 95% confidence intervals were (-0.066, -0.017) for high DAP and (-0.109, -0.027) for low DAP, and the difference bootstrap 95% confidence intervals were (0.002, 0.064), indicating the presence of moderated mediating effects. Similarly, the difference in the indirect effects was significant when patient-oriented OCB was the dependent variable. In summary, H5a and H5b are supported.

**Table 7:** Conditional process analyses

Mechanism		Effect	BootSE	BootLLCI	BootULCI
PM-EE-SP	High DAP (+ 1 SD)	-0.039	0.013	-0.066	-0.017
	Low DAP (-1 SD)	-0.063	0.020	-0.109	-0.027
	Difference	0.024	0.015	0.002	0.064
PM-EE-OCB	High DAP (+ 1 SD)	-0.040	0.014	-0.123	-0.023
	Low DAP (-1 SD)	-0.065	0.025	-0.072	-0.015
	Difference	0.025	0.017	0.002	0.070

*PM* patient mistreatment, *DAP* displaced aggression by patients, *EE* emotional exhaustion, *SP* service performance, *OCB* patient-oriented organizational citizenship behavior

**Fig. 3:**
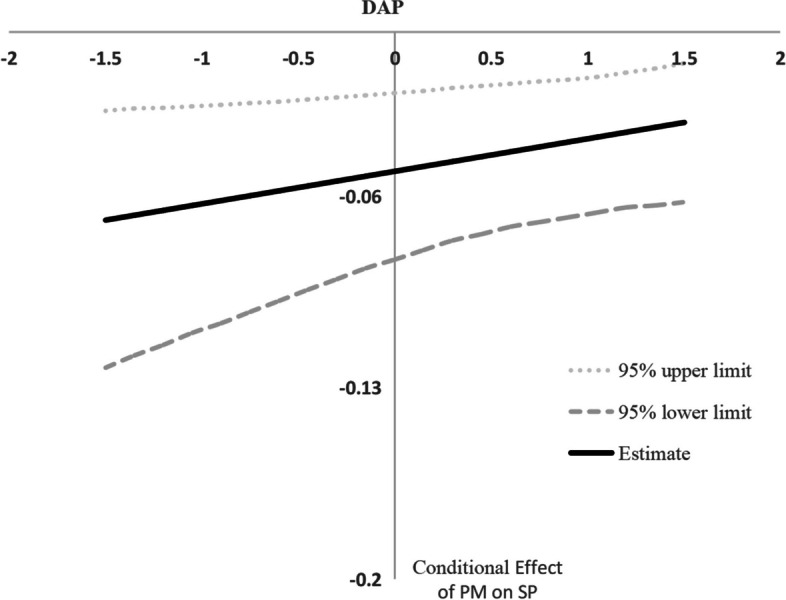
Conditional effect of PM on SP as a function of DAP. Note: PM, patient mistreatment; DAP, displaced aggression by patients; SP, service performance

**Fig. 4:**
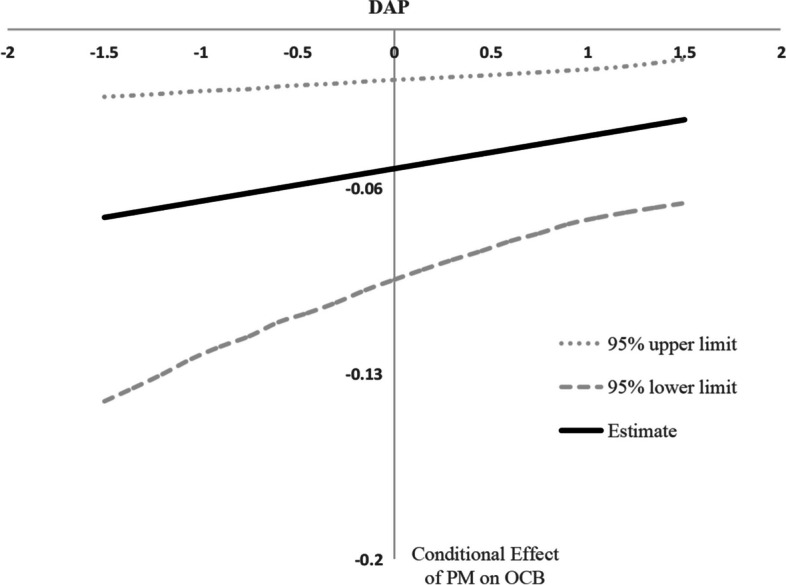
Conditional effect of PM on OCB as a function of DAP. Note: PM, patient mistreatment; DAP, displaced aggression by patients; OCB, patient-oriented organizational citizenship behavior

Finally, to overcome the limitation that moderated mediation can only be tested for discrete moderator values (namely, mean and mean ± one standard deviation) [64], we used the Johnson-Neyman method to depict the continuous confidence intervals of indirect effects. Figures 3 and 4 visualized the moderating mediation effect of different DAP levels (after standardization), indicating that the effect of patient mistreatment on service performance and patient-oriented OCB through emotional exhaustion was weaker when perceived displaced aggression by patients was higher.

## Discussion

This study was among the first to reveal the relationship between patient mistreatment and healthcare workers’ role behaviors in the context of the Fangcang shelter hospital. The positive emotions and attitudes of healthcare workers are conducive to their work performance [65, 66]. Existing research suggests that patient mistreatment not only affect the mood of healthcare workers [67] but also impede patient safety [68]. It may also reduce work productivity and performance [69, 70]. Given the lack of related studies on patient mistreatment in Fangcang shelter hospitals and the call by Caldas et al. (2021) to study the negative effects of public health emergencies on healthcare workers [71], we investigated the impacts of patient mistreatment on healthcare workers' in-role and extra-role behaviors in the Chinese fight against COVID-19.

Using the conservation of resources theory and self-verification theory, this study demonstrated that patient mistreatment led to exhaustion of the mental resources of healthcare workers, which in turn reduced their service performance and patient-oriented OCB. This process was moderated by healthcare workers' perceived DAP. The effect of patient mistreatment on the emotional exhaustion of healthcare workers was greater, and the indirect effect of mistreatment on the two types of role behaviors via emotional exhaustion was stronger when DAP was low versus high. This study identified the pathway and boundary condition by which patient mistreatment influences service performance and patient-oriented OCB, and verified all the hypotheses of the study.

Our study is consistent with previous work showing that patient mistreatment has a negative impact on emotional exhaustion in healthcare workers [72, 73]. Employees often consume their own emotional resources after being mistreated by customers, resulting in psychological distress, reducing service quality and increasing turnover intention [74]. Based on the above findings, we further explored the impacts of patient mistreatment on workers’ service performance and patient-oriented OCB. Previous studies have mainly documented the negative effects of customer mistreatment in the service industry. Few studies have examined the effects on healthcare workers' behaviors within shelter hospitals during the epidemic [75]. The special environment of shelter hospitals requires healthcare workers to interact with patients with the highest frequency and to have a high level of empathy. Empathetic professionals are better able to place themselves in the patient’s position, thereby making them less likely to reduce their own serving efforts [76].

The present study innovatively considered a moderating variable, namely, displaced aggression by patients. Previous research on DAP has primarily focused on antecedents and outcome variables. DAP was used in this study to illustrate the boundary condition behind the link between patient mistreatment and healthcare workers’ role behaviors, emphasizing the importance of preserving their self-efficacy after perceiving DAP. It allows healthcare workers to attribute mistreatment to patient-directed blame, such as patients’ anger, depression and other negative emotions, which results in indiscriminate attacks against the staff, rather than to their own failure to meet medical service goals. From the perspective of empathy, it also explains how patient mistreatment influences service performance and patient-oriented OCB via emotional exhaustion and how this relationship changes with displaced aggression.

### Limitations and future directions

This study has several limitations. First, self-reports were used to measure the key constructs. Studies have shown good validity of the self-report measures used in this study [77]. However, the data collection was based on the overall perceptions of healthcare staff in Fangcang shelter hospitals. In the future, changes in healthcare workers’ emotional states could be explored by using a more detailed longitudinal research design or contextual experimental approach. Second, shelter hospitals are relatively narrow and confined environments in which patients tend to "indiscriminately" attack healthcare workers. Frontline healthcare workers who understand the psychological stress and negative emotions of patients are more inclined to regulate their emotions to meet the needs of patients when encountering mistreatment. Future research can be conducted in other contexts to better understand the antecedents and outcomes of patient mistreatment and develop appropriate strategies to address it in different healthcare scenarios.

## Conclusions

In summary, the COVID-19 has caused public panic and psychological stress. Anxiety and depressive symptoms are common psychological reactions during epidemics [78]. Patients in Fangcang shelter hospitals, due to isolation time, economic income losses, sealed environment and other factors, may develop acute stress disorder, anxiety, fear, insomnia and other psychological problems. We need to improve health promotion education and psychological guidance for patients in hospitals to alleviate the various negative emotions brought about by the pandemic and patients' aggression against healthcare workers.

Although healthcare workers in shelter hospitals have a higher capacity of empathy, they are at 3–4 times higher risk of being infected with the virus than others because of their long-term exposure to the infected population [79]. Lai et al. (2020) found that half of the healthcare workers working in Wuhan Fangcang shelter hospitals had more severe psychological symptoms due to fear of being infected [80]. Moreover, suffering from severe mistreatment by patients exacerbates the emotional exhaustion of medical personnel. It is important to pay attention to the dynamic optimization of healthcare staff shifts and provide early psychological interventions for those with psychological problems. In addition, training them to express empathy is imperative for the effective operation of shelter hospitals. When interacting with patients, workers may be required to have skills such as negotiation, stress management, and mindfulness. Hospitals need to assist the staff in learning these skills and putting them into practice. If employees are financially or non-financially compensated for being mistreated by patients, the negative impact on their service behavior may be reduced.

## Data Availability

The data that support the findings of this study are available on request from the corresponding author.

## Abbreviations

OCB: Organizational citizenship behavior
DAP: Displaced aggression by patients
